# Endoscopic Treatment of Colonic Duplication Cyst: A Case Report and Review of the Literature

**DOI:** 10.1155/2018/6143570

**Published:** 2018-02-13

**Authors:** Rajat Garg, Louis D. Saravolatz, Mohammed Barawi

**Affiliations:** ^1^Department of Internal Medicine, St. John Hospital and Medical Center, Detroit, MI, USA; ^2^Department of Medicine, St. John Hospital and Medical Center, Detroit, MI, USA; ^3^Division of Gastroenterology and Hepatology, St. John Hospital and Medical Center, Detroit, MI, USA

## Abstract

Colonic duplication cysts are a rare congenital abnormality commonly presenting before two years of age. In adults, it has been rarely reported, most often as an incidental finding. We report a case of 42-year-old female complaining of constipation and lower abdominal pain. Patient's CT scan of the abdomen showed a cystic lesion at hepatic flexure and the diagnosis was confirmed endoscopically using endoscopic ultrasound (EUS). The cyst was treated employing hot snare to expose the cyst cavity. On our literature search, there have been no reported cases of colonic duplication cyst treated endoscopically. We here discuss incidence, diagnosis, characteristics, and treatment of duplication cysts with special emphasis on endoscopic therapy.

## 1. Introduction

Duplication cyst was first described by Fitz in 1884 for the remnant of omphalomesenteric duct [1]. They are rare congenital cysts commonly presenting before two years of age and rarely reported in adults. We report a case of colonic duplication cyst diagnosed endoscopically and treated using endoscopic ultrasound (EUS).

## 2. Case Report

**Figure 1:**
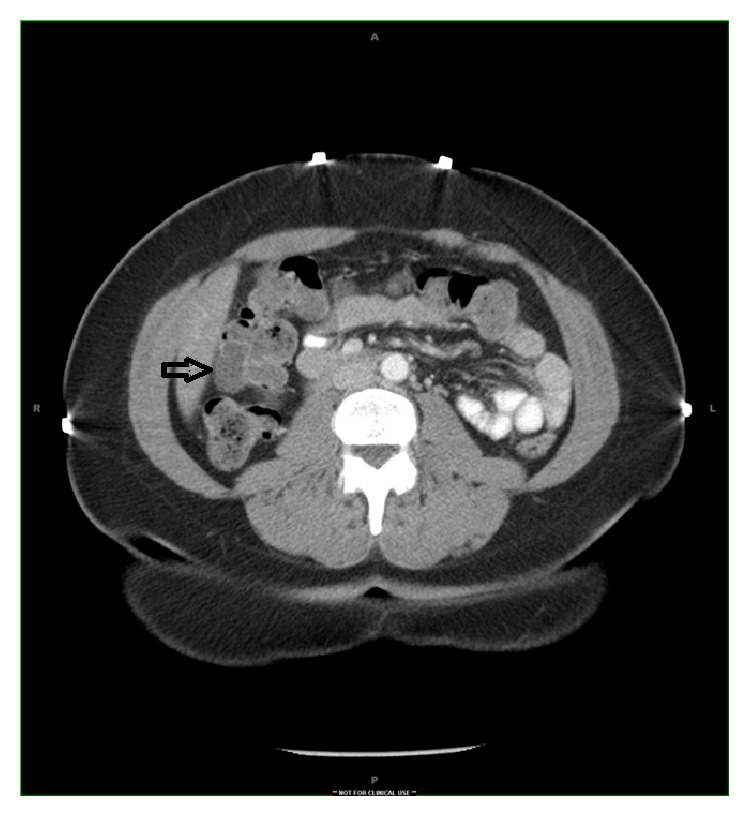
Computed tomographic image showing moderately enhancing cystic lesion at hepatic flexure (arrow).

A 42-year-old female with no past medical history presented with the complaints of constipation and lower abdominal pain for four months. There was no history of anorexia, weight loss, or hematochezia. Examination was negative for any masses or organomegaly. The patient had colonoscopy performed which was negative for any polyps and showed a large submucosal lesion at the hepatic flexure. The lesion showed vascular congestion and was tattooed with India ink. The patient was then referred to an advanced endoscopist for further evaluation. Computed tomography (CT) scan of the abdomen with contrast revealed a 20 mm moderately enhancing cystic lesion at the hepatic flexure (Figure 1).

**Figure 2:**
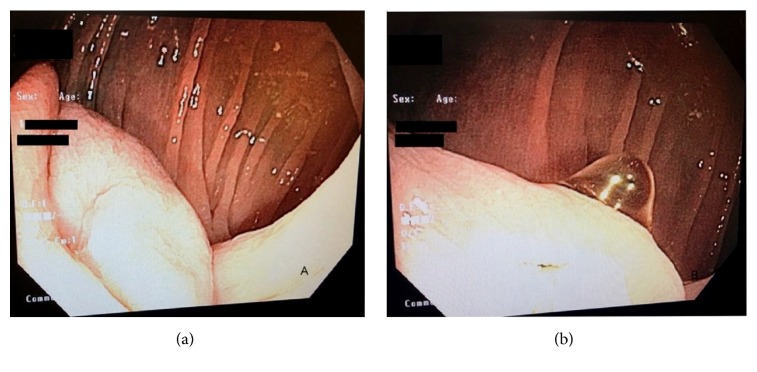
Intraluminal mass at hepatic flexure (a) and transparent appearance (b).

**Figure 3:**
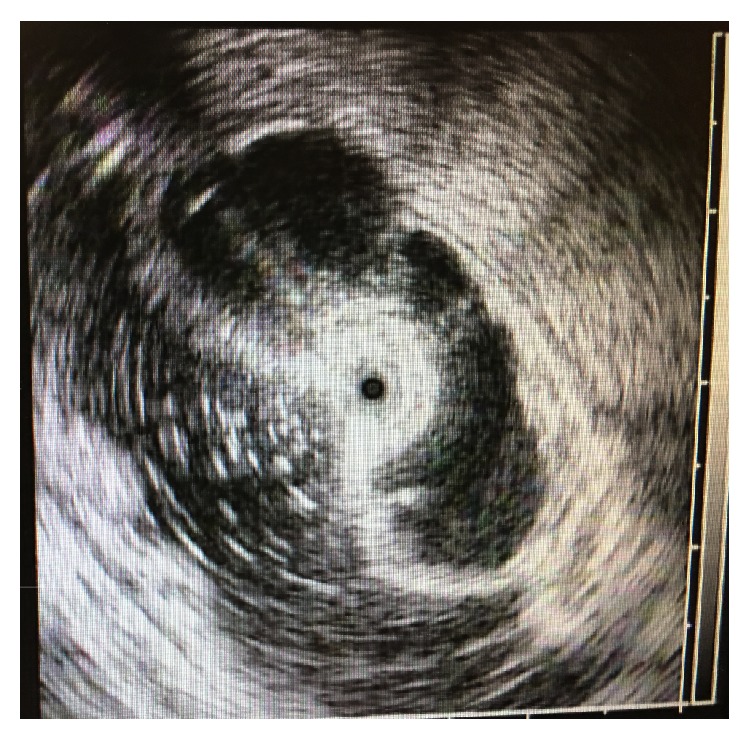
12 MHz EUS images showing subepithelial anechoic lesion lined by mucosa.

**Figure 4:**
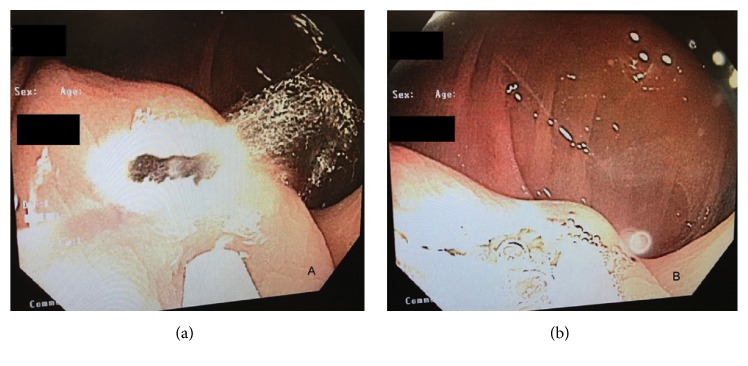
Fluid drainage after unroofing the cyst cavity (a) and flattening of lesion after drainage (b).

**Figure 5:**
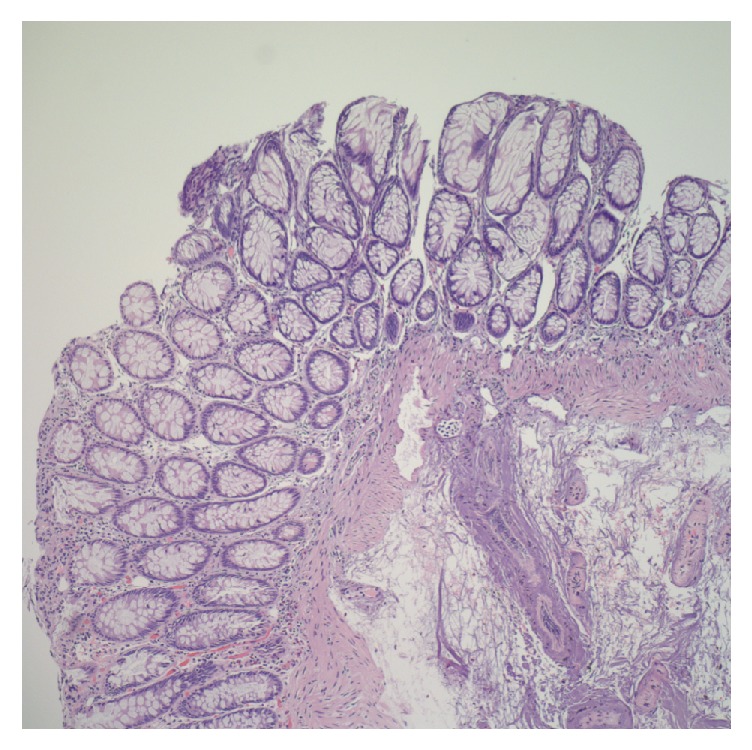
Normal colonic mucosa with thermal cautery artifact.

On repeat colonoscopy, a tattooed subepithelial intraluminal transparent mass lesion was seen at the hepatic flexure (Figure 2). A 12 MHz mini-EUS probe was passed through the biopsy channel of the colonoscope which revealed a 14 mm subepithelial anechoic cystic lesion lined by mucosa consistent with colonic duplication cyst (Figure 3). Hot snare was used to unroof and expose the cyst cavity (Figure 4(a)). Clear fluid was seen gushing from the mass with flattening of the lesion after drainage (Figure 4(b)). The patient had an uneventful recovery and was discharged home with outpatient follow-up. Pathologic evaluation showed normal colonic mucosa without any dysplastic changes (Figure 5).

## 3. Discussion

Colonic duplication cysts are a rare entity encountered in the pediatric age group and even less common in adults. They are hollow, epithelium lined, cystic, spherical, or tubular structures attached to gastrointestinal tract. Several theories have been postulated regarding their occurrence but no single theory can explain all the duplication cysts [2]. There are four theories to explain the development of duplication cysts: aberrant luminal recanalization theory as suggested by Bremer, the diverticular theory of Lewis and Thyng, the split notochord theory that explains the formation of neurenteric duplications and associated vertebral anomalies, and the intrauterine vascular accident theory which is also the most accepted one postulating that vascular accidents in utero are responsible for these anomalies. Its actual incidence is unknown due to the rarity of publications of this disease but its estimated incidence is 1 in 4500 [3].

Duplication cysts can occur anywhere from the oral cavity to the rectum. They are named according to the location in the gastrointestinal tract. In the largest case series of 96 pediatric patients, the most common site was ileum (35%) followed by esophagus (21%) and colon (20%) [2]. Another case series that reported only 6.8% of total duplication cyst involves colon [4]. Male sex is more affected than females with a ratio of 1.5 : 1. Caucasians (80%) are more affected than African Americans (20%).

Duplication cysts can be cystic (80%) or tubular (20%) and commonly occur on the mesenteric side of bowel sharing a common blood supply with native bowel. The majority of duplication cysts communicate with bowel with only a few cases reported of noncommunicating duplication cyst [5]. Most adult colonic cysts are asymptomatic and remain undiagnosed over many years; however, the most common presentation is abdominal pain [2, 4, 6, 7]. Cases of duplication cysts causing volvulus, intussusception, rectal bleeding, ischemic bowel, constipation, and obstruction have been described in the literature [8–10]. Due to such variable presentations, diagnosis of duplication is rarely made preoperatively and often made intraoperatively or on pathologic examination.

The wall of duplication cysts is comprised of two layers: the outer muscular layer and the inner mucosal layer. Duplication cysts can also have ectopic tissue of two origins most commonly heterotopic gastric mucosa, which can ulcerate leading to bleeding and less commonly pancreatic tissue [6]. Ectopic gastric tissue has been reported in case series with an incidence of 2–5% [2, 4, 7]. In addition, malignant degeneration has been reported to occur in duplication cysts most commonly in adults. Malignant degeneration has been reported in few case reports and can be anywhere from dysplasia to metastatic adenocarcinoma [11, 12]. Two cases of squamous cell cancer arising from these cysts have been reported in the literature [13]. Despite few reports of malignancy arising from colonic duplication cysts, it still remains extremely rare and only discovered incidentally. In the largest case series of duplication cysts in adults, dysplastic changes were found in 2 of 7 patients: one patient had high-grade dysplasia, and the other patient had low-grade dysplasia [7].

Despite the advancement of imaging modalities, diagnosis of duplication cysts is difficult and often made postoperatively on pathologic examination. Ultrasound (US) and CT scan are commonly used in making a diagnosis of duplication cyst. US may reveal a fluid filled complex cyst with internal echoes. Duplication cysts are characterized by typical inner echogenic mucosa and outer hypoechoic muscular layer. On US, the presence of peristalsis in the cyst is an important feature as it is not seen in any other cystic lesions. Contrast studies will demonstrate a filling defect or communication with the bowel. CT scan may reveal a cystic mass with rarely reported calcified focus [14]. As in our case, colonoscopy with EUS can assist in diagnosing duplication cyst if other studies are normal and the cyst is communicating with the colon. EUS has been reported as safe and accurate for diagnosis of duplication cysts and is commonly employed for diagnosing duodenal and foregut duplication cysts but rarely used for colonic duplication cysts [15]. The typical appearance of duplication cyst on sonographic examination is a “muscular rim sign” manifesting as inner echogenic mucosa and outer hypoechoic muscle layer; however, these changes are sometimes not present due to some degree of involution and degeneration [16]. If a typical inner echogenic mucosal and outer hypoechoic muscle layer can be seen on US, the diagnosis of duplication cyst can be established. In our case, EUS revealed an anechoic lesion lined by mucosa leading to the diagnosis of duplication cyst.

There are no standardized guidelines or consensus for the treatment of duplication cysts. Surgical resection is classically done for the cyst as they are found incidentally or intraoperatively [4, 16]. Duodenal duplication cysts have long been effectively treated endoscopically commonly using needle knife papillotomy with reported good long-term outcomes [17, 18]. Resection of the cyst roof using a snare is sufficient to relieve the symptoms. Due to the concerns of malignancy and incidental diagnosis, en bloc surgical resection is usually recommended for the colonic duplication cyst; however, endoscopic therapy can effectively treat patient's symptoms while avoiding potential extensive surgery and its related complications as surgical resection often involves resection of some part of native bowel due to cyst's intimate attachment with the bowel. One must be cautious in concluding that there is no malignancy with a biopsy of the cystic endothelium alone and further surveillance must be incorporated to determine if malignancy is still present. There are less than twenty cases reported in English language of malignant changes associated with colonic duplication cysts [14]. Moreover, avoiding irritation from cyst secretions theoretically can prevent dysplastic degeneration. Random biopsies should be done to rule out potential premalignant and malignant changes and, if possible, inner layer of the cyst should be biopsied as well. Endoscopic surveillance with random biopsies after 6 and 12 months of treatment as recommended for duodenal duplication cyst should be done to monitor the response to therapy and any premalignant or malignant changes [18].

Our patient had EUS findings consistent with a colonic duplication cyst and the biopsy was negative for any dysplasia; thus effective drainage was established using hot snare resulting in resolution of the patient's symptoms. There is a chance that malignant cells might be missed on biopsy, if they were present in cystic endothelium and the drainage secretion. For the above reason, we reiterated the role of endoscopic surveillance every 6 months for at least one year. There is no data on extended follow-up. If there is any abnormal pathology on regular follow-up in first year, then multidisciplinary and individualized approach should be taken. If the pathology is negative for any malignant/dysplastic changes in first year, there is low likelihood of malignant transformation in the future; however, caution should be exercised in case any gastrointestinal symptoms appear or recur. From our literature search, there has been no reported case of colonic duplication cyst treated endoscopically. EUS guided drainage of colonic duplication cyst is an effective approach for diagnosis and treatment of colonic duplication cysts provided that the lesion can be easily accessed and random biopsies can be done to rule out any premalignant or malignant changes. This case highlights the potential of endoscopic therapy for the diagnosis and management of colonic duplication cysts and may be considered to avoid potentially extensive surgery.
